# Occupational Therapy, Self-Efficacy, Well-Being in Older Adults Living in Residential Care Facilities: A Randomized Clinical Trial

**DOI:** 10.3389/fpsyg.2018.01414

**Published:** 2018-08-07

**Authors:** Abel Toledano-González, Teresa Labajos-Manzanares, Dulce María Romero-Ayuso

**Affiliations:** ^1^Department of Psychology, Faculty of Occupational Therapy, Speech Therapy and Nursing, University of Castilla-La Mancha, Talavera de la Reina, Spain; ^2^Department of Physical Therapy, Faculty of Health Sciences, University of Málaga, Málaga, Spain; ^3^Division of Occupational Therapy, Department of Physical Therapy, Faculty of Health Sciences, University of Granada, Granada, Spain

**Keywords:** human activity, occupational therapy, personal satisfaction, self-efficacy, well-being

## Abstract

**Introduction:** Choosing the type of treatment approach is as important as the treatment itself, also giving and important value to internal variables in the individual that can determine the evolution of the intervention. The main aim of this study is to determine whether individual and/or group occupational therapy leads to changes in generalized self-efficacy and psychological well-being, and to identify the type of therapy that has the best effects on older adults.

**Method:** Prospective, randomized, comparing two treatment groups: individual and group therapy during 6 months. A total sample of 70 patients institutionalized in residential care homes for older adults with a mean age of 85 (*SD* = 4). Assessment was conducted using the *General Self-Efficacy Scale and Ryff’s Well-being Scale*. For analyze the main dependent variables we used ANOVA for intra-subject and inter-subject factors and Pearson correlation between well-being and self-efficacy by type of treatment.

**Results:** Groups were equivalent at baseline. The results show statistically significant differences between the two types of therapy, showing a positive correlation between well-being and self-efficacy, being greater at a group level than at and individual level. At the group level, practically all of variables measured in the participants were increased as shown in the results tables, including a better adaptation and predisposition to work four participants died while the study was being conducted.

**Conclusion:** The clinical trial shows that older people in residential centers achieve an increase in emotional well-being and self-efficacy when they receive occupational therapy group, rather than individual treatment not being significant changes. Treatment group participants reported a positive experience and clinical benefits from training program.

The clinical trial was registered in the U.S. National Institutes of Health (ClinicalTrials.gov) with NCT02906306 identifier.

## Introduction

Nowadays, there are many older people who find it difficult to carry out the activities of daily life normally, but in many cases assessments focus exclusively on the functional level, leaving aside psychological factors, especially the sense of competence and how it can influence the successful implementation of any type of activity we want to develop (Kirby et al., 2015). The design and analysis of activities therapeutic in occupational therapy can facilitate the creation of models of life satisfaction. It is necessary to study carefully the demands the activity requires of an individual, depending on their needs, competences and interests, in order to achieve the highest possible level of occupational adaptation in each particular case, thus promoting rewarding aging (Kielhofner, 2007; Shearer and Guthrie, 2013; Holmberg and Ringsberg, 2014). The aging process differs for every individual, depending on their experiences and how they interpret and reconstruct them. The aging is a process that progresses steadily, except for when a traumatic situation suddenly generates drastic changes (Calenti, 2010; Kim et al., 2012; Voigt-Radloff et al., 2013).

The psychological well-being has been defined as the balance between expectations, hopes, dreams, possible or achieved realities, which are expressed through satisfaction and the capacity to cope with life events in order to successfully adapt to daily life (Molina and Meléndez, 2006). Ryff describes psychological well-being as a subjective, multidimensional construct, which can be defined by the individual as the sense and meaning of one’s own life (Ryff and Singer, 2002; Ortiz and Castro, 2009).

Regarding the sense and meaning of human activity in emotional well-being, one of the emerging theories is that which Csikszentmihalyi proposes, that one way to understand activities regards the adaptation or alignment of an individual’s capacities and the demands of the task at hand, producing a sensation of flow or optimal experience. Csikszentmihalyi’s flow theory is applicable in occupational therapy, when determining the focus of treatment and the time to switch activity so as to provide an optimal experience through the performance of tasks, drawing on the user’s capacities and skills, establishing a balance between the possibilities of completing the activity and the individual’s capacity to undertake it (Csikszentmihalhyi, 2009). However, the quality of the experience is a fundamental factor to be taken into account, either individually or in a group way, for the feeling perceived intrinsically by the person and from the activity, perceiving a balance between personal skills and the activity as a challenge (Stavrou et al., 2015).

Taking into account how they can influence the psychological variables to the adults González and Extremera (2010) analyzed the association between optimism, self-esteem, performance of activities of daily living (ADLs) and well-being in older adults. They found a low to moderate correlation between well-being and participation in social activities, mass communication use and activities outside home. Their results also showed that self-esteem relates significantly with social and creative activities, becoming an interesting association to analyze the relationship between self-esteem and type of activity. Furthermore, they found a positive relationship between optimism and social, leisure and free-time activities. Social cognitive theory defends self-efficacy as an influence on successful performance, as well as the observation of success in other people, making the stimulus very valuable and whose appropriate interpretation can suppose a positive experience (Giesbrecht and Miller, 2017).

Another of the variables analyzed in the study that is directly influenced is self-efficacy. Can be defined as a set of beliefs about one’s capabilities to organize and perform the courses of action needed to realize certain achievements or desired results. The level of an individual’s self-esteem is a key consideration for promoting occupational performance (Hermida and Stefani, 2012). There exists a relationship between performing an activity, understood as a set of actions undertaken to reach a goal (adapted to the skills and capacities of the user), self-efficacy (way of thinking, feeling or acting) and well-being (set of factors which form part of the user’s quality of life) (Zimmer et al., 1995).

The relationship between self-efficacy and self-esteem may be diminished by lack of activity, resulting in a feeling of low self-efficacy (Aguirre Mas and Vauro Desiderio, 2009). Broadly speaking, between the ages of 25 and 65, the feeling of competence or self-efficacy is more robust, since in this period individuals present optimal levels of activity, with enhanced and diversified performance of roles. However, from the age of 65, individuals gradually withdraw from these roles and their associated activities. Hence, they are more vulnerable to feelings of low self-efficacy and personal competence, and to a decline in psychological well-being. This has been explained by the social disengagement theory formulated by Cummings and Henry (1961), which claims that older adults’ disengagement from society is a result of the normal aging process and mutual withdrawal (Brodaty et al., 2010). The literature reports the existence of a large number of people with problems of low self-efficacy, prevalence of depression, which leads to a state of low quality of life and well-being, contributing to lower rates of recruitment and retention (Corcoran et al., 2016). Two types of intervention have always been used, individual and group, but it has never been measured to what extent the person can benefit from the psychological variables that type of intervention can report better results. The main objective of this study is to determine whether individual and/or group occupational therapy leads to changes in generalized self-efficacy and psychological well-being, and to identify the type of therapy that has the best effects on the population of older adults. In addition, we aim to determine whether there is a relationship between the different domains of psychological well-being and the sense of general self-efficacy. There are trends that recognize well-being as the absence of negative conditioning factors that can influence important well-being, are very important in gerontology. Therefore, staying positive and maintaining a positive feeling is recognized as a positive feeling toward a positive aging, becoming indicators related to self-efficacy and well-being (Triadó et al., 2007).

## Materials and Methods

### Study Procedure

The research draws on a randomized experimental intervention study with a pre-post design, comparing two types of treatment: individual and group therapy.

The clinical trial was conducted at two state-assisted residential care centers for older adults in Málaga (Spain) who agreed to participate in the study of those selected, there is no differences in interesting between the two residential care centers. All participants was first interviewed to gather sociodemographic data (age, gender, education level) as well as to verify that their scores for cognitive levels and the required skills and capabilities were sufficient to participate in the study, in order to carry out without difficulty the activities to be carried out. The participants were assessed through the MMSE (Mini-Mental Status Examination) (Lobo et al., 2002) needing a higher score than 22. During this interview, the aim and length of the intervention was explained and informed consent was obtained before enrolment in the trial. The clinical trial was registered in the U.S. National Institutes of Health (ClinicalTrials.gov) with NCT02906306 identifier and approved with 01/2012 registration by the University of Málaga Ethics Committee. All participants gave their written consent.

The planning study and sample selection occurred along months of January 2015 to March 2015 (6 months), for the Pre-Test measured throughout the month of April, after the sample was selected, participants were randomized to two groups of 37. Following assessment, the intervention began in May to end of October. After that, participants were re-assessed for the Post-Test along months of November 2015 to January 2016, the statistical treatment along March 2016 to December 2016.

### Participants

From an initial sample of 112 residents, a final sample of 74, divided into two groups of 37, was included in the study. **Table 1** (see below) shows the sociodemographic characteristics of the sample. For both groups, the inclusion criteria were: (1) able to read; (2) having normal cognitive function, scoring > 22 on the Mini Mental Scale Examination, the cut-off point according to the scoring instructions of the adapted version for the Spanish population (Miquel and Agustí, 2011). The exclusion criteria established were health conditions that contraindicate or prevent treatment such as hearing loss, fear of animals, acute visual impairment, intermittent claudication or repeated failure to perform during the study.

**Table 1:** Age and baseline data of the two groups.

						CI (95%)	

		Ind. TRT	Group TRT	*P-*value	Differences between groups	Lower limit	Upper limit
Age^∗^	Mean (*SD*)	85.22 (4.00)	85.57 (4.04)	0.860	-0.171	-2.106	1.763
Sex^∗^	Mean (*SD*)	1.22 (0.417)	1.27 (0.450)	0.594	-0.054	-0.255	0.147
Education level^∗^	Mean (*SD*)	1.51 (0.731)	1.65 (0.716)	0.424	-0.135	-0.470	0.200
GSE^∗^		24.43 (5.532)	24.23 (5.30)	0.878	0.200	-2.389	2.789
Ryff^∗^	A	20.29 (4,390)	19.94 (4.158)	0.738	1.022	-1.697	2.382
	PR	22.17 (1.932)	21.91 (1.704)	0.557	0.436	-0.612	1.126
	Auton.	27.49 (4.591)	26.77 (3.797)	0.481	1.007	-1.295	2.724
	EM	20.63 (1.942)	20.37 (1.911)	0.578	0.460	-0.662	1.176
	PG	24.97 (1.902)	24.37 (2.045)	0.208	0.472	-0.342	1.542
	PL	20.83 (3.601)	20.40 (3.310)	6.06	0.827	-1.221	2.078


*A, self-acceptance; PR, positive relations; Auton., autonomy; EM, environmental mastery; PG, personal growth; PL, purpose in life.*

*GSE, General Scale Efficacy).*

*^∗^2 certificate death not included in each group.*

### Intervention

The activities were conducted in three 45-min sessions per week. In both treatment modalities the activities included personal independence training (ADLs), sensory-motor stimulation activities, cognitive stimulation (attention, memory, language, and executive function) and animal-assisted therapy (AAT). The group occupational therapy participants also received psychosocial skills training.

Participants were assigned in four subgroups, three of them were formed by nine patients and the last one was created by 10 persons, in each treatment for working more easily and comfortable, since they were too many to put together in a single group.

Within the group of individual occupational therapy, the patient works independently to other patients and without social interaction, enhancing capabilities such as attention or concentration or specific activities that need continuous supervision, improving their individual effectiveness. Individual therapy focuses on individual aspects of the person, patient-centered, maximizing ability to function autonomously without help through a positive feedback to improve confident, self-efficacy and subjective experience deficits (Waters et al., 2012; Fletcher-Smith et al., 2013).

Nevertheless, in occupational therapy group patients work together, increasing levels of efficiency through the stimulation group experiences and problems are shared, developing a relationship between them, making the therapist a driver of the activity throughout the activity. This allows the feedback is greater, actively working in the direction and the process of the activity (Kayama et al., 2014; Nagayama et al., 2015).

### Outcomes

The primary outcome was the well-being, assessed using the Ryff’s psychological wellbeing scale, adapted to Spanish by Van Dierendock (Díaz et al., 2006) consisting of 39 items to be rated on a scale of 1 to 6, 1 indicating strong disagreement and 6 indicating strong agreement. In its original version, this scale includes six dimensions: self-acceptance, purpose in life, positive relations with others, autonomy, environmental mastery, and personal growth. All of them present a good internal consistency, with α Cronbach levels equal or higher than 0.70, supporting it’s valid use for older adults (Tomás Miguel et al., 2008)

There is a short version that reduces the original subscales to four: (1) self-acceptance; (2) interpersonal relations; (3) autonomy; and (4) life satisfaction. This new version successfully represents the basic theoretical elements of each dimension, reduces the length of the original scales and, at the same time, improves the psychometric properties of most of the existing English versions (Díaz et al., 2006).

The secondary outcome was the self-efficacy assessed using the General self-efficacy scale (GSE) by Schwarzer and Jerusalem (1995) adapted to Spanish (Baessler and Schwarcer, 1996). It is a 10-item self-reported scale, designed to assess general self-efficacy with a Likert 1–4 format, in which 1 stands for strong disagreement, and 4 strong agreements. It comprises a great variety of situations, and it has been translated to many languages. There is evidence of its construct validity, reliability test–retest, and its internal consistency (α Cronbach 70%) (Sanjuán Suárez et al., 2000).

### Statistical Methods

To describe the sample, we used descriptive statistics for quantitative and qualitative variables. To analyze differences in the main study variables we applied the Student’s *t*-test or the Mann–Whitney *U* test, depending on whether or not they followed a normal distribution. Chi square testing was used to determine the existence of differences in distribution by gender, age and educational level. To analyze the main dependent variables (self-efficacy and well-being) we conducted a repeated measures ANOVA for intra-subject and inter-subject factors. Statistical significance was established at *p* < 0.05. Data were analyzed using SPSS, version 21.0.

The study sample size of 74 participants was determined with EPIDAT 4.0. in a mean comparison of matched samples with a power (1-beta) of 80%, and a significance level of 95%, a d/s of 0.5, with a total of 65 subjects per matched sample, which nine subjects were added for possible losses.

According with calculations of the sample size for hypothesis with a power of 80%, it has been estimated for 74 participants would need in total, 37 in each group (see **Table 1**). The patients were randomized into either the individual therapy or group therapy. Participants were randomly assigned following simple randomization procedures, throughout STATS software, to individual or group treatment. Data were collected by only one occupational therapist in both centers specialized in geriatrics (single blind).

## Results

**FIGURE 1:**
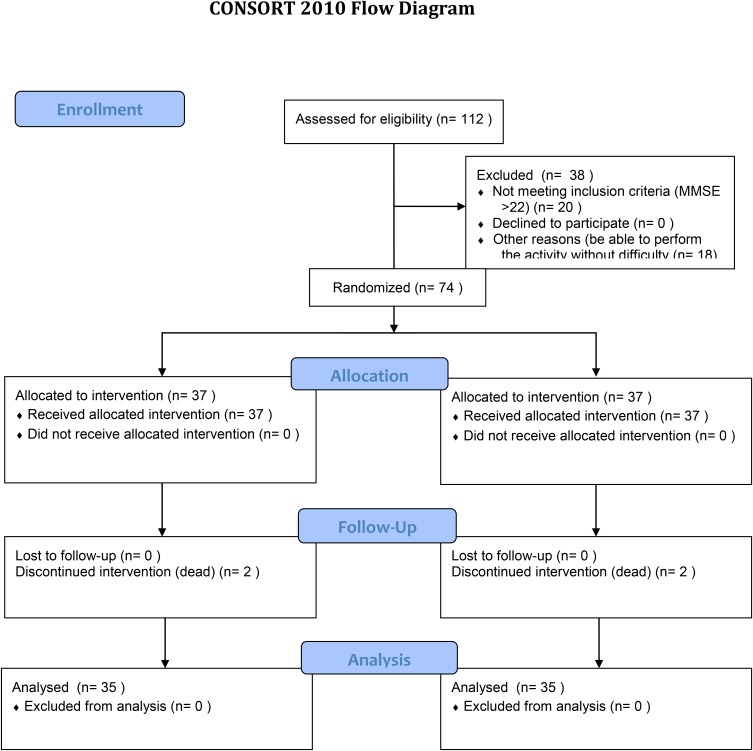
Consort flow diagram for participant recruitment.

Along intervention four persons did not finish the intervention, reducing the total sample to 70 participants, due to death causes, two in each group. The distribution by type of activity is uniform between men and women in both groups (*p* = 0.569), aged between 78 and 95 years (*p* = 0.709), with an average of 85 years among groups. About sex, clear difference between women (77.1%) and men (22.9%). The educational level was comparable in the two groups, in both groups only there were 13.5% with 4-year degree or higher; for individual therapy 24.2% had high school graduate or equivalent while 37.8% for group therapy; 62.3% had not primary school diploma for individual therapy while 48.7% had not any diploma in group therapy (*p* = 0.400). There are no differences in the assignment of the sample with the sociodemographic data. A CONSORT flow diagram for recruitment is provided in **Figure 1**.

Although initially we found no statistically significant differences in the different domains of psychological well-being among the participants in both groups (Pre-Test), the results for psychological well-being generally showed the positive effect of the group occupational therapy (Post-Test). Perceived psychological well-being was higher in the group occupational therapy than in the individual occupation therapy. With the individual therapy methodology, we only found statistically significant differences in two domains of emotional well-being, namely self-acceptance (*p* = 0.004) and positive relations with others (*p* = 0.003) (see **Table 2**).

**Table 2:** Results for Emotional Well-Being and Self-Efficacy post-treatment.

	Individual Mean (*SD*)	Grupal Mean (*SD*)	*t*	*P-*value	Differences between groups	CI 95%		F Intra-subjects				F Inter-subjects	

						Lower limit	Upper limit	Ind		Group		*F*	*p*
										
								*t*	*p*	*T*	*p*		
**GSE**	16,43 (4,520)	32,74 (5,622)	-13.380	<0.001	-16.314	-18.747	-13.881	10.841	<0.001	-8.169	<0.001	179.016	<0.001
**Self-acceptance**	18,91 (17,33–20,50)	27,09 (25,73–28,44)	-7.550	<0.001	-8.171	-10.221	-6.122	3.118	0.004	-9.338	<0.001	63.289	<0.001
**Positive relations with others**	20,20 (18,56–21,84)	26,89 (25,44–28,33)	-6.210	<0.001	-6.686	-8.834	-4.538	3.202	0.003	-7.267	<0.001	38.569	<0.001
**Autonomy**	27,03 (25,28–28,78)	33,14 (31,62–34,67)	-5.352	<0.001	-6.114	-8.394	-3.835	0.906	0.371	-8.134	<0.001	28.641	<0.001
**Environmental mastery**	21,37 (20,36–22,38)	26,23 (25,03–27,43)	-6.296	<0.001	-4.857	-6.397	-3.318	-1.687	0.101	-10.858	<0.001	39.634	<0.001
**Personal growth**	25,00 (24,08–25,92)	31,00 (29,59–32,41)	-7.237	<0.001	-6.000	-7.654	-4.346	-0.059	0.954	-8.846	<0.001	52.372	<0.001
**Purpose of life**	20,91 (19,73–22,10)	27,63 (26,33–28,92)	-7.777	<0.001	-6.714	-8.437	-4.992	-0.241	0.811	-10.196	<0.001	60.487	<0.001


*GSE, General Scale Efficacy.*

The results for self-efficacy yielded statistically significant differences between the two types of therapy (*p* < 0.001). Significant differences were also found in the intra-subject measures in both group occupational therapy (*p* < 0.001) and individual occupational therapy (*p* < 0.001) (see **Table 2**). However, only group occupational therapy enhanced the sense of self-efficacy in the older adults. It is important to underline that the total self-efficacy was lower after individual occupational therapy (*p* < 0.001) (see **Table 3**).

**Table 3:** Results for the correlation between Emotional Well-Being and Self-Efficacy by treatment type.

Individual Therapy		Post A	Post GSE	Post PR	Post Auton	Post EM	Post PC	Post PL
	**Post A**	1	0.549^∗∗^	n.s.	0.646^∗∗^	0.505^∗∗^	n.s.	0.838^∗∗^
	**Post GSE**		1	n.s.	n.s.	n.s.	n.s.	0.486^∗∗^
	**Post PR**			1	0.586^∗∗^	n.s.	0.427^∗^	n.s.
	**Post Auton.**				1	0.388^∗∗^	n.s.	0.603^∗∗^
	**Post EM**					1	n.s.	0.547^∗∗^
	**Post PC**						1	0.356^∗^
	**Post PL**							1
**Group Therapy**								
	**Post A**	1	0.806^∗∗^	0.867^∗∗^	0.779^∗∗^	0.702^∗∗^	0.655^∗∗^	0.886^∗∗^
	**Post GSE**		1	0.786^∗∗^	0.746^∗∗^	0.585^∗∗^	0.615^∗∗^	0.845^∗∗^
	**Post PR**			1	0.771^∗∗^	0.696^∗∗^	0.635^∗∗^	0.751^∗∗^
	**Post Auton.**				1	0.742^∗∗^	0.596^∗∗^	0.758^∗∗^
	**Post EM**					1	0.491^∗∗^	0.632^∗∗^
	**Post PC**						1	0.665^∗∗^
	**Post PL**							1


*A, self-acceptance; PR, positive relations; Auton, autonomy; EM, environmental mastery; PG, personal growth; PL, purpose in life.*

*GSE, General Scale Efficacy.*

*^∗∗^Correlation is significant at 0.01.*

*^∗^Correlation is significant at 0.05.*

The results of the correlation between emotional well-being and self-efficacy by treatment type shows that in the individual therapy we only found a statistically significant correlation between self-efficacy (*p* < 0.001) and self-acceptance and purpose in life (*p* = 0.003). By contrast, in the group therapy we found a statistically significant correlation between all the domains of psychological well-being and sense of self-efficacy (see **Table 3**).

## Discussion

Our results show that the older adults receiving group occupational therapy improved in all the domains of psychological well-being and in sense of self-efficacy (GSE). By contrast, the older adults receiving individual occupational therapy only improved in the domain of environmental mastery and their scores for self-acceptance and positive relations with others decreased, which leads us to think that individual treatment diminished self perception by lowering self-acceptance scores and positive relationships, reducing the amount of feedback that is perceived in the performance of an activity. In addition, their sense of self-efficacy declined. The results obtained in the between-groups comparison (individual vs. group) are in concordance with the findings of Zimmer (Zimmer et al., 1995). This supports the possible relationship between life satisfaction and psychological well-being, suggesting that group therapy provides greater reinforcement and greater variety of feedback than individual therapy (Kayama et al., 2014). Similarly, Andonian and MacRae (2001) found a relationship between well-being and health promoted by social participation among older adults and their perception of competence, caused by the loss of social roles.

The results obtained from the individual therapy group with those of the study by García-Campayo et al. (2005) who finds that the mere fact of participating in treatment leads to enhanced efficacy in emotional expression, social relations, and emotional lability (Liddle and Eagles, 2014).

However, we found no changes in intra-subject results for the individual therapy intervention, which were highly similar to pre- and post-intervention results. Following individual intervention, results remained the same for the variables of autonomy, personal growth and purpose in life (see **Table 2**). The fact that only the group treatment modality produces changes could be explained by the sense of positivity produced by the commitment to completing a task, which in turn promotes enhancements in the subscales of autonomy, environmental mastery and personal growth. This emotional response, mentioned by Csikszentmihalhyi (2009) strengthens commitment and a sense of well-being, which our results demonstrate.

The results on the general self-efficacy scale suggest that group occupational therapy promotes a greater feeling of efficacy, thus providing participants with a bigger sense of emotional support, enhancing and individual’s perception of competence thorough the use of feedback from a group will increase not only the perception of health but also general well-being, making the social factor a variable to be taken into account whenever possible in the implementation of an intervention (Waters et al., 2012; Fletcher-Smith et al., 2013). These data suggest that well-being and self-efficacy (Andonian and MacRae, 2001) are bolstered by the sense of belonging to a group. The emotional support generated by social participation not only enhances general well-being, but also self-concept and self-efficacy, associated with an increase in social activity (Zimmer et al., 1995; Green and Harvey, 2014). The feeling of commitment to a group increases the sense of self-efficacy and the communication involved facilitates well-being and promotes the health of older adults, who feel strengthened by the sense of commitment and social connection, thus achieving a sense of higher self-efficacy (Herranz Aguayo et al., 2013; Shimada et al., 2016).

Arguably, the decline in self-efficacy and psychological well-being generated in some of the variables for the older adults receiving individual therapy, is due to the patient being more aware of their limitations, while in group therapy this effect is mitigated and greater feedback is received. Furthermore, the significant positive correlation between self-efficacy and self-acceptance could be explained by the fact that the latter refers to how individuals feels good about themselves despite being aware of their own limitations (Díaz et al., 2006). Additionally, purpose in life, which refers to defining objectives to give a meaning to life, may be affected by the sense of efficacy. Older adults may feel less competent, and thus find it difficult to set new objectives that give sense to life.

Psychological well-being increased among participants in group occupational therapy while results for those receiving individual occupational therapy remained stable across some dimensions (autonomy, personal growth, and purpose in life) and even declined in others (self-acceptance and positive relations). Statistically significant differences were found in all inter-subjects and between-groups measures (see **Table 2**).

With regard to general self-efficacy, group occupational therapy was more effective than individual occupational therapy. Inter-subject and between-group tests yielded statistically significant data in both groups but with the reverse result (see **Table 2**). The results for participants in group therapy show an enhancement in self-efficacy, while the older adults in the individual therapy group exhibit a decline.

The relationship between self-efficacy and well-being is found to be directly proportional, that is, the greater the sense of well-being, the higher the level of self-efficacy. These enhanced scores are found in the group occupational therapy, which clearly exhibits a greater impact on older adults, demonstrating the connection between self-efficacy and well-being, with regard to an individual’s general mood state and greater life satisfaction.

### Methodological Considerations

This study has a series of limitations. First, the work should arguably have been conducted with a larger sample, although the required sample size was calculated for a study with two comparison groups of similar characteristics to ours.

Second, we did not consider the presence of multiple pathologies, which might have benefitted either one of the treatment modalities. However, given that participants were randomized to groups, we feel the any potential effect would arguably have been equally distributed across the groups.

Third, a possible limitation is the time it took to carry out the post-test, in some cases taking up to 3 months, even so the data are still significant maintaining it in time the effect of the therapy.

Lastly, the inclusion of a third, non-treatment control group might have shed more light on the impact of occupational therapy on institutionalized older adults.

## Conclusion

In conclusion, the results from the current study confirm that the *g*roup occupational therapy generated positive effects with statistically significant differences on the psychological well-being and general self-efficacy of institutionalized older adults, in contrast to the results of individual occupational therapy providing older people with a significant improvement in scores of their general self-efficacy, psychological well-being versus individual occupational therapy. Participant’s perceived benefit of the intervention was high through the optimal experience and group activities. An interesting future research direction is to verify whether similar results are generated in other types of non-residential populations, for example, day-care centers, hospital outpatients or sheltered housing.

## Author Contributions

AT-G: concept and design, acquisition of subjects, analysis, interpretation of data, and preparation of manuscript. DMR-A: concept and design, analysis, interpretation of data, and preparation of manuscript. TL-M: concept and design, analysis, interpretation of data, and preparation of manuscript.

## Conflict of Interest Statement

The authors declare that the research was conducted in the absence of any commercial or financial relationships that could be construed as a potential conflict of interest.
